# On the Use of Large Catheters

**Published:** 1811-09

**Authors:** 


### *To the Editors of the Medical and Physical Journal.*

Gentlemen,

I WAS rather surprised in perusing your Journal for April, 1809, page 314, to find the following remark of Dr. Roberton on Spasmodic Stricture: he says, "We are certainly much indebted to Mr. Home for having introduced more generally into practice the use of the large in preference to the small catheter." Mr. Sharp, however, in page 183 of his Critical Enquiry, was rather before him in his opinion respecting the comparative advantage of a large over a small instrument. He says, "a large catheter or sound may sometimes be passed into the bladder when a small one cannot." But that Mr. Sharp was not the first who perceived the superior advantage in many instances of a large in preference to a small catheter, will appear evident from the following passage in the second part of Heister's Institutions of Surgery, where he observes, "Some approve of their being very small, or slender, thinking that thereby they have a more easy passage into the bladder, in which they are much mistaken; because the most slender ones are apt rather to catch and stick in the rugæ and inequalities of the urethra, which often appear very considerable in old men, so that the whole operation may be thereby frustrated, &c. &c." The above quotation is sufficient to sanction what I have advanced, that Mr. Home was not the first discoverer of the superior superior advantage in many instances of the large catheter; possibly he was unacquainted with the above passage, if so, he claims equal merit with Heister.

I am, &c. &c.

B. E. T.

*Hythe, July* 12, 1811.

